# An OGT-STAT5 Axis in Regulatory T Cells Controls Energy and Iron Metabolism

**DOI:** 10.3389/fimmu.2022.874863

**Published:** 2022-07-08

**Authors:** Zengdi Zhang, Oscar C. Salgado, Bing Liu, Zahra Moazzami, Kristin A. Hogquist, Michael A. Farrar, Hai-Bin Ruan

**Affiliations:** ^1^ Department of Integrative Biology and Physiology, University of Minnesota, Minneapolis, MN, United States; ^2^ Center for Immunology, University of Minnesota, Minneapolis, MN, United States; ^3^ Department of Food Science and Nutrition, University of Minnesota, Minneapolis, MN, United States; ^4^ Department of Laboratory Medicine and Pathology, University of Minnesota, Minneapolis, MN, United States

**Keywords:** O-GlcNAc, Treg - regulatory T cell, iron, fatty acid uptake, adipose tissue

## Abstract

The immunosuppressive regulatory T (Treg) cells exert emerging effects on adipose tissue homeostasis and systemic metabolism. However, the metabolic regulation and effector mechanisms of Treg cells in coping with obesogenic insults are not fully understood. We have previously established an indispensable role of the O-linked N-Acetylglucosamine (O-GlcNAc) signaling in maintaining Treg cell identity and promoting Treg suppressor function, *via* STAT5 O-GlcNAcylation and activation. Here, we investigate the O-GlcNAc transferase (OGT)-STAT5 axis in driving the immunomodulatory function of Treg cells for metabolic homeostasis. Treg cell-specific OGT deficiency renders mice more vulnerable to high-fat diet (HFD)-induced adiposity and insulin resistance. Conversely, constitutive STAT5 activation in Treg cells confers protection against adipose tissue expansion and impaired glucose and insulin metabolism upon HFD feeding, in part by suppressing adipose lipid uptake and redistributing systemic iron storage. Treg cell function can be augmented by targeting the OGT-STAT5 axis to combat obesity and related metabolic disorders.

## Introduction

Foxp3^+^ regulatory T (Treg) cells are specialized immunosuppressive lymphocytes that control self-tolerance, inflammatory responses, and tissue homeostasis (1, 2). Treg cells develop in the thymus through two distinct programs involving CD25^+^Foxp3^-^ and CD25^-^Foxp3^lo^ progenitors (3). The upregulation of CD25 during Treg cell development is driven by strong T cell receptor (TCR) signals (4). The upregulation of Foxp3 is TCR-independent, but instead involves IL-2 signaling through its receptor CD25 and the downstream activation of STAT5 (5–7). Besides of their indispensability during development, both TCR and IL-2/STAT5 signaling pathways are required for the suppressive function of effector Treg cells (8–10). Treg cells are essential in enforcing peripheral tolerance and loss of them leads to a fatal scurfy phenotype that is characterized by severe inflammation in endocrine and barrier organs (11–13).

In addition to preserving tissue integrity, Treg cells have been increasingly shown to support adipose tissue function and systemic metabolism. Treg cells, along with other anti-inflammatory immune cells, are abundant in adipose tissues of lean animals (14, 15). The accumulation and function of adipose Treg cells depend on antigen presentation by parenchymal MHC class-II molecules and local cytokines such as IL-33 to drive a PPARγ-dependent transcriptional program (16–18). Obesity profoundly remodels the immune cell compartment of fat tissue, leading to Treg cell reduction and insulin resistance. Adoptive transfer or genetic induction of Treg cells have been shown to suppress adipose inflammation and improve insulin sensitivity in obese animals (19–21). On the other hand, loss of Treg cell function by cell depletion, PPARγ knockout, or Vα5/Vβ8.2 TCR transgene leads to no or marginal changes in adipose insulin resistance (14, 18, 22). It has been proposed that Treg cells in obesity and aging may adopt pro-inflammatory properties (15, 23); therefore, restoring Treg cell function, rather than increasing Treg cell number, may offer therapeutic opportunities for metabolic diseases. Nonetheless, mechanisms mediating the insulin-sensitizing effect of adipose Treg cells are not fully understood. Also, it has not been established if Treg cells can directly modulated lipid metabolism and energy balance.

O-linked N-Acetylglucosamine (O-GlcNAc) modification at serine or threonine residues, termed O-GlcNAcylation (24), plays a central role in signaling pathways relevant to chronic human diseases such as diabetes, cancer, and immune disorders (25–27). O-GlcNAc transferase (OGT), using UDP-GlcNAc derived from the hexosamine biosynthetic pathway as the substrate, controls diverse biological processes such as gene transcription, protein stability, and cell signaling (28–31). O-GlcNAcase (OGA) mediates the removal of O-GlcNAcylation from proteins. Protein O-GlcNAcylation senses glucose availability (30, 32), hormonal cues (29, 33, 34), cellular stress (33, 35), and immune and bacterial signals (27, 36, 37). Recently, we found in Treg cells that TCR signaling promotes OGT-mediated O-GlcNAcylation of Foxp3 and STAT5, two transcriptional factors essential for Treg cell development and function (38). Overexpression of a constitutive active form of STAT5 rescues lineage instability and suppressor dysfunction found in O-GlcNAc deficient Treg cells, thus ameliorating systemic autoimmunity in OGT-deficient mice (38). In this study, we seek to interrogate roles of the OGT-STAT5 axis in instructing Treg cells to cope with obesity-associated remodeling of the adipose tissue and systemic metabolism.

## Materials and Methods

### Mice

*Foxp3^YFP-cre^* mice (stock number 016959) and *Foxp3^eGFP-Cre-ERT2^* mice (stock number 016961) were purchased from the Jackson Laboratory. *Rosa26^Stat5b-CA^* mice were provided by Dr. Alexander Rudensky at Memorial Sloan Kettering Cancer Center (10). *Ogt^fl/fl^* Mice (Jackson Laboratory stock number 004860) were kindly provided by Dr. Xiaoyong Yang at Yale University. All mice were on C57BL/6 background. Mice were free to access water and fed on a regular chow, tamoxifen food (Teklad, TD.130860), or 60% high fat diet (ResearchDiets, D12492) as indicated. All procedures were approved by the Institutional Animal Care and Use Committee at the University of Minnesota. All relevant ethical regulations for animal testing and research were complied with.

### Metabolic Assays

Body weights were monitored every week. Body composition was assessed using an EchoMRI system (39). For food intake measurement, mice were individually housed and food consumption was weighed every morning for 7 consecutive days. For the metabolic cage study, mice were first acclimated in metabolic chambers (Columbus Instruments), and then physical activity and energy expenditure were measured continuously for at least 2 days (40). For oral glucose-, and insulin- tolerance tests. 16 h fasted mice were given glucose (1.5 g/kg body weight) by gavage; 6 h fasted mice were injected with insulin (1 U/kg body weight) intraperitoneally. Tail-vein blood collected at the designated times was used to measure blood glucose level using a Contour Glucometer (Bayer). For lipid tolerance test, mice were fasted for 4 h, gavaged with 200 ul of olive oil, and tail-vein blood was collected every 1.5 h for quantification of triglyceride using a colorimetric assay kit (Cayman #10010303). Fecal energy was determined with a bomb calorimeter at Dr. Pedro Urriol’s lab. Fecal lipid was extracted with chloroform: methanol mix (41), weighed and calculated as percentage to fecal weight.

### Flow Cytometry

For surface markers, cells were stained in PBS containing 0.5% BSA with relevant antibodies at 4°C for 30 min. For analysis of intracellular markers, cells were first fixed with Fixation/Permeabilization buffer (ThermoFisher, catalog no. 00-5123) at 4°C for 30 min and then stained in Permeabilization Buffer (ThermoFisher, catalog no. 00-8333) with relevant antibodies at 4°C for 30 min. Flow cytometry data were acquired on BD Fortessa H0081 or X-20 and analyzed with Flowjo.

### Histology

Mouse tissues were dissected and fixed in 10% buffered formalin. Sectioning and haematoxylin & eosin (H&E) staining were performed by the Comparative Pathology Shared Resource of UMN.

### RNA and Real-time PCR

RNA was isolated with Trizol and reverse transcribed into cDNA with the iScript™ cDNA Synthesis Kit. Real-time RT-PCR was performed using iTaq™ Universal SYBR^®^ Green Supermix and gene-specific primers on a Bio-Rad C1000 Thermal Cycler. All data were normalized to the expression of *18s* or *36b4.*

### Western Blotting

Tissues were lysed in buffer containing 1% Nonidet P-40, 50 mM Tris 3 HCl, 0.1 mM EDTA, 150 mM NaCl, proteinase inhibitors and an OGT inhibitor, TMG. Equal amounts of protein lysates were electrophoresed on 4-20% SDS-PAGE gels and transferred to Nitrocellulose membranes. Primary antibodies were incubated at 4°C overnight. Western blotting was visualized by peroxidase conjugated secondary antibodies and ECL chemiluminescent substrate with a Bio-Rad ChemiDoc Imaging System or imaged by fluorescent IgG secondary antibodies with a LI-COR Odyssey 9120 imager.

### Statistical Analyses

Results are shown as mean ± SEM. The comparisons were carried out using two-tailed Student’s t test, one-way ANOVA followed by multiple comparisons with the Tukey adjustment, two-way ANOVA using GraphPad Prism 7.

## Results

### OGT-insufficiency in Treg Cells Leads to Insulin Resistance

**Figure 1:**
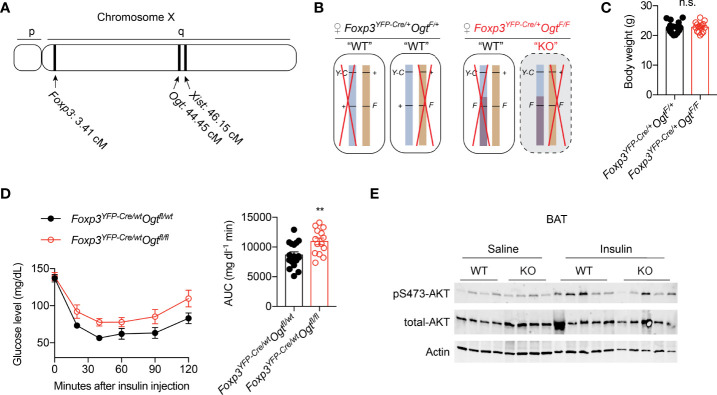
Mosaic OGT-deficiency in Treg cells decreases insulin sensitivity. **(A)** Chromosome positions of mouse *Foxp3* and *Ogt* genes. The *Xist* gene is shown for reference. cM, centimorgan. **(B)** Schematic view of allele combinations of the *Foxp3* and *Ogt* genes in *Foxp3^YFP-Cre/wt^Ogt^F/+^* and *Foxp3^YFP-Cre/wt^Ogt^F/F^* female mice. *Y-C*, *YFP-Cre*; *F*, floxed; +, wildtype (wt). Red “X” indicates random inactivation of one of the two X chromosomes. **(C)** Body weight of 3-month-old, NC-fed *Foxp3^YFP-Cre/wt^Ogt^F/+^* (n = 18) and *Foxp3^YFP-Cre/wt^Ogt^F/F^* (n = 15) female mice. **(D)** Insulin tolerance test of 3-month-old, NC-fed *Foxp3^YFP-Cre/wt^Ogt^F/+^* (n = 17) and *Foxp3^YFP-Cre/wt^Ogt^F/F^* (n = 14) female mice. Area under curve (AUC) is shown to the right. **(E)** pS473-Akt and total-Akt immunoblotting of BAT protein lysates from *Foxp3^YFP-Cre/wt^Ogt^F/+^* and *Foxp3^YFP-Cre/wt^Ogt^F/F^* female mice treated with saline or insulin for 30 min. Data are presented as mean ± SEM. n.s., not significant; **, p < 0.01 by unpaired student’s t-test.

Both the *Foxp3* and *Ogt* genes are located on the mouse X Chromosome; about 40 centimorgans apart (**Figure 1A**). When we generated Treg cell-specific OGT knockout (KO) using the *Foxp3^YFP-Cre^* mice with YFP-Cre knocked into the endogenous *Foxp3* locus (**Figure S1A**), hemizygous male KO mice developed scurfy phenotypes and died of autoimmune disease by the age of 4 weeks (38), thus preventing us to study their whole-body metabolism. Due to random inactivation of the X chromosome in females, *Foxp3^YFP-Cre/wt^Ogt^F/F^* female mice possess both OGT-sufficient (WT) and -deficient (KO) Treg cells (**Figure 1B**). Despite lineage instability and suppressor dysfunction of KO Treg cells, the remaining WT cells were sufficient to avert autoimmunity in mice (38). Therefore, *Foxp3^YFP-Cre/wt^Ogt^F/F^* females and their littermate controls were used to investigate metabolic outcomes. When fed with normal chow (NC), no difference in body weight was observed in 12-week-old mice (**Figure 1C**). Compared to controls, *Foxp3^YFP-Cre/wt^Ogt^F/F^* females had higher blood glucose levels during insulin tolerance test, indicative of impaired insulin sensitivity (**Figure 1D**). Glucose tolerance test revealed similar blood glucose levels between the two genotypes (**Figure S1B**), suggesting that pancreatic insulin secretion might be increased to compensate insulin resistance in KO mice. We then treated mice with insulin to assess its downstream signaling, and found similar AKT phosphorylation in gonadal white adipose tissue (gWAT) and liver (**Figure S1C**). However, insulin-stimulated Ser473 phosphorylation of AKT in brown adipose tissue (BAT) was diminished in *Foxp3^YFP-Cre/wt^Ogt^F/F^* females (**Figure 1E**). These results demonstrate that OGT in Treg cells controls insulin pathway activation in BAT and whole-body insulin sensitivity.

### OGT-insufficiency in Treg Cells Promotes Diet-induced Obesity

**Figure 2:**
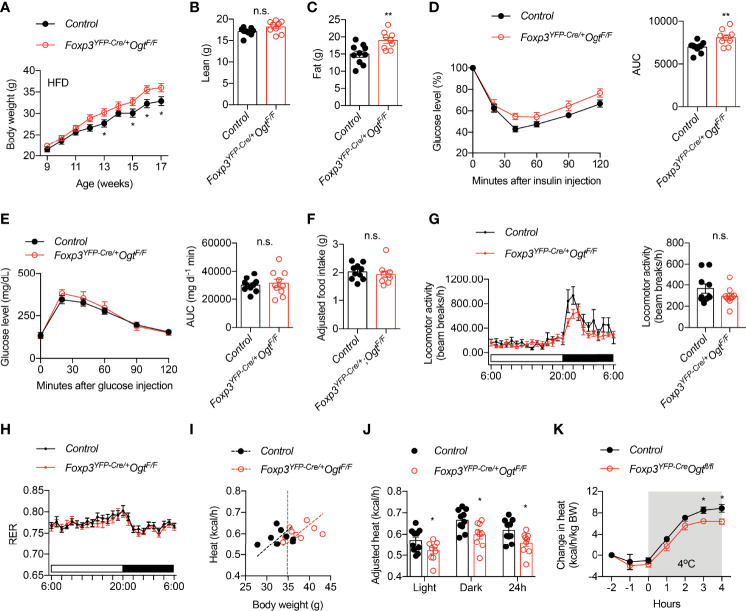
*Foxp3^YFP-Cre/wt^Ogt^F/F^* mice are prone to diet-induced obesity. **(A)** Growth curve of *Foxp3^YFP-Cre/wt^Ogt^F/+^* (n = 19) and *Foxp3^YFP-Cre/wt^Ogt^F/F^* (n = 17) female mice after HFD feeding. **(B)** Lean mass and **(C)** fat mass of HFD-fed *Foxp3^YFP-Cre/wt^Ogt^F/+^* (n = 10) and *Foxp3^YFP-Cre/wt^Ogt^F/F^* (n = 9) female mice. **(D)** Insulin tolerance test of HFD-fed *Foxp3^YFP-Cre/wt^Ogt^F/+^* (n = 10) and *Foxp3^YFP-Cre/wt^Ogt^F/F^* (n = 9) female mice. Area under curve (AUC) is shown to the right. **(E)** Glucose tolerance test of HFD-fed *Foxp3^YFP-Cre/wt^Ogt^F/+^* (n = 10) and *Foxp3^YFP-Cre/wt^Ogt^F/F^* (n = 10) female mice. Area under curve (AUC) is shown to the right. **(F)** Daily HFD intake of *Foxp3^YFP-Cre/wt^Ogt^F/+^* (n = 10) and *Foxp3^YFP-Cre/wt^Ogt^F/F^* (n = 9) female mice. ANCOVA was used to correct food intake for body weight. **(G–J)** Metabolic cage study of HFD-fed *Foxp3^YFP-Cre/wt^Ogt^F/+^* (n = 10) and *Foxp3^YFP-Cre/wt^Ogt^F/F^* (n = 9) female mice showing locomotor activity **(G)**, respiratory exchange ratio (RER, H), and heat **(I)**. Body weight-adjusted heat analyzed with ANCOVA is shown **(J)**. **(K)** Cold-induced change in heat production of HFD-fed *Foxp3^YFP-Cre/wt^Ogt^F/+^* (n = 5) and *Foxp3^YFP-Cre/wt^Ogt^F/F^* (n = 5) female mice. Data are presented as mean ± SEM. n.s., not significant; *, p < 0.05; **, p < 0.01 by unpaired student’s t-test **(B–G, J)** or two-way ANOVA **(A, K)**.

We then fed animals with 60% high fat diet (HFD) to induce obesity. Female *Foxp3^YFP-Cre/wt^Ogt^F/F^* mice gained more weight than controls (**Figure 2A**). Body composition analysis showed that weigh gain was due to increased fat mass (**Figures 2B, C**). Similar to animals fed with chow, HFD *Foxp3^YFP-Cre/wt^Ogt^F/F^* females displayed impaired insulin tolerance (**Figure 2D**), but normal glucose tolerance (**Figure 2E**).

We went on to determine what caused diet-induced adiposity in *Foxp3^YFP-Cre/wt^Ogt^F/F^* mice. Their daily intake of HFD was similar to control mice, when adjusted to body weight using ANCOVA analysis (**Figure 2F**). Metabolic cage study found no difference in locomotor activity (**Figure 2G**) or respiratory exchange ratio (**Figure 2H**). Rather, heat production, when adjusted to body weight (42), was significantly reduced in *Foxp3^YFP-Cre/wt^Ogt^F/F^* mice during both light and dark periods (**Figures 2I, J**). Moreover, when challenged with cold, *Foxp3^YFP-Cre/wt^Ogt^F/F^* animals showed reduced heat production than the controls (**Figure 2K**), indicative thermogenic defects. Together, these data demonstrate that OGT-controlled Treg cell function enables metabolic adaptation to positive energy balance.

### STAT5 Activation in Treg Cells Confers Protection Against Diet-induced Obesity

**Figure 3:**
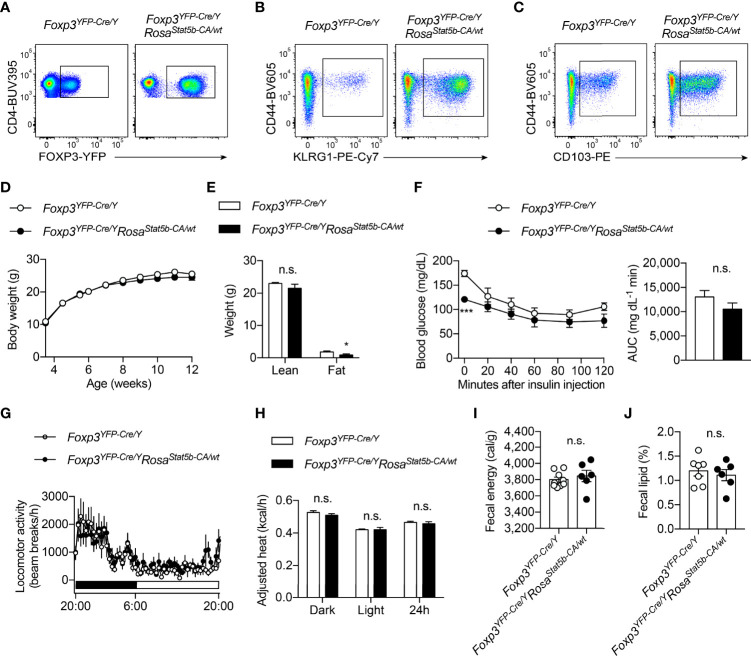
Characterization of *Foxp3^YFP-Cre/Y^Rosa26^Stat5b-CA/wt^* mice. **(A–C)** Flow cytometric quantification of FOXP3-YFP^+^CD4^+^ Treg cells **(A)**, CD44^+^KLRG1^+^ **(B)** and CD44^+^CD103^+^ **(C)** effector Treg cells in lymph nodes from *Foxp3^YFP-Cre/Y^* and *Foxp3^YFP-Cre/Y^Rosa26^Stat5b-CA/wt^* mice. **(D)** Growth curve of *Foxp3^YFP-Cre/Y^* (n = 10) and *Foxp3^YFP-Cre/Y^Rosa26^Stat5b-CA/wt^* (n = 9) mice fed with NC. **(E)** Lean and fat mass of NC-fed *Foxp3^YFP-Cre/Y^* (n = 7) and *Foxp3^YFP-Cre/Y^Rosa26^Stat5b-CA/wt^* (n = 6) mice. **(F)** Insulin tolerance test of NC-fed *Foxp3^YFP-Cre/Y^* (n = 8) and *Foxp3^YFP-Cre/Y^Rosa26^Stat5b-CA/wt^* (n = 7) mice. Area under curve (AUC) is shown to the right. **(G)** Locomotor activity and **(H)** body weight-adjusted heat production of NC-fed *Foxp3^YFP-Cre/Y^* (n = 7) and *Foxp3^YFP-Cre/Y^Rosa26^Stat5b-CA/wt^* (n = 6) mice, determined by metabolic cages. **(I)** Fecal energy and **(J)** fecal lipid percentage of NC-fed *Foxp3^YFP-Cre/Y^* (n = 7) and *Foxp3^YFP-Cre/Y^Rosa26^Stat5b-CA/wt^* (n = 6) mice. Data are presented as mean ± SEM. n.s., not significant; *, p < 0.05; ***, p < 0.001 by unpaired student’s t-test or two-way ANOVA **(D, F)**.

The regulation of Treg cell suppressive program by OGT is dependent on STAT5 O-GlcNAcylation and activation (38). We then sought to determine whether STAT5 overactivation, to the opposite of OGT knockout, would drive Treg cell action in systemic metabolism. *Foxp3^YFP-Cre/Y^Rosa26^Stat5b-CA/wt^* male mice were generated to specifically overexpress a constitutively active (CA-) form of STAT5B in Treg cells (10). Flow cytometric analyses confirmed that STAT5B-CA not only increased the number of FOXP3^+^ Treg cells in the lymph nodes and the activity of the *Foxp3* gene promoter (**Figure 3A**), but also drastically expanded the population of effector Treg cells that were CD44^+^KLRG1^+^ (**Figure 3B**) or CD44^+^CD103^+^ (**Figure 3C**). When fed with a normal chow, even though with comparable body weight (**Figure 3D**), *Foxp3^YFP-Cre/Y^Rosa26^Stat5b-CA/wt^* mice showed a slight, but statistically significant reduction in fat mass (**Figure 3E**). There was a declining trend of blood glucose levels during the insulin tolerance test (**Figure 3F**). Similar locomotor activity (**Figure 3G**), energy expenditure (**Figure 3H**), and energy or lipid excretion into the feces (**Figures 3I, J**) were observed between control and *Foxp3^YFP-Cre/Y^Rosa26^Stat5b-CA/wt^* mice.

**Figure 4:**
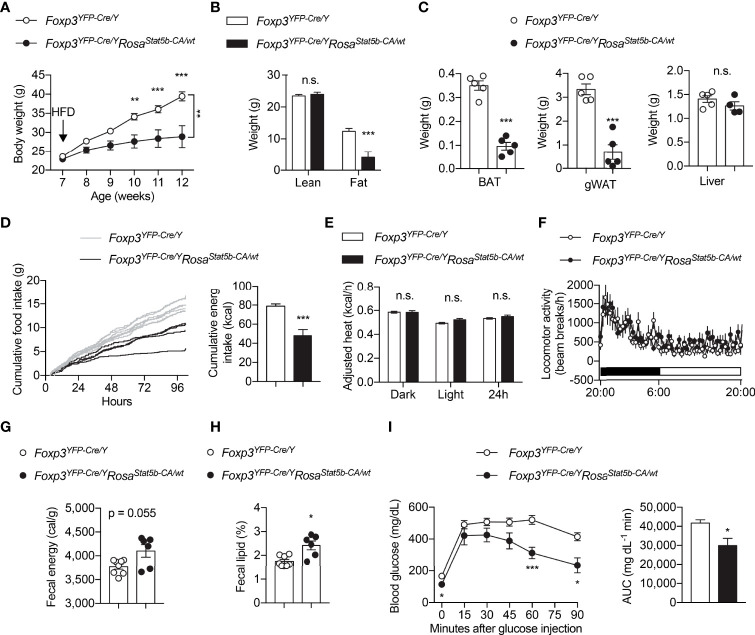
Protection of *Foxp3^YFP-Cre/Y^Rosa26^Stat5b-CA/wt^* mice from diet-induced obesity. **(A)** Body weight of *Foxp3^YFP-Cre/Y^* (n = 9) and *Foxp3^YFP-Cre/Y^Rosa26^Stat5b-CA/wt^* (n = 6) mice after HFD feeding. **(B)** Lean and fat mass of HFD-fed *Foxp3^YFP-Cre/Y^* (n = 9) and *Foxp3^YFP-Cre/Y^Rosa26^Stat5b-CA/wt^* (n = 6) mice. **(C)** Weight of BAT, gonadal WAT, and liver of HFD-fed *Foxp3^YFP-Cre/Y^* (n = 5) and *Foxp3^YFP-Cre/Y^Rosa26^Stat5b-CA/wt^* (n = 5) mice. **(D)** Cumulative food intake of *Foxp3^YFP-Cre/Y^* (n = 7) and *Foxp3^YFP-Cre/Y^Rosa26^Stat5b-CA/wt^* (n = 4) mice when switched to HFD feeding. **(E)** Body weight-adjusted heat production and **(F)** locomotor activity of HFD-fed *Foxp3^YFP-Cre/Y^* (n = 11) and *Foxp3^YFP-Cre/Y^Rosa26^Stat5b-CA/wt^* (n = 6) mice, determined by metabolic cages. **(G)** Fecal energy and **(H)** fecal lipid percentage of HFD-fed *Foxp3^YFP-Cre/Y^* (n = 8) and *Foxp3^YFP-Cre/Y^Rosa26^Stat5b-CA/wt^* (n = 6) mice. **(I)** Glucose tolerance test of HFD-fed *Foxp3^YFP-Cre/Y^* (n = 9) and *Foxp3^YFP-Cre/Y^Rosa26^Stat5b-CA/wt^* (n = 6) mice. Area under curve (AUC) is shown to the right. Data are presented as mean ± SEM. n.s., not significant; *, p < 0.05; **, p < 0.01; ***, p < 0.001 by unpaired student’s t-test or two-way ANOVA **(A, I)**.

We went on to challenge the mice with HFD. The resistant to HFD-induced weight gain could be readily seen in *Foxp3^YFP-Cre/Y^Rosa26^Stat5b-CA/wt^* mice as early as 3 weeks after diet-switching (**Figure 4A**). The reduction in weight gain was solely because of reduced fat mass (**Figure 4B**). Both BAT and gWAT depots were significantly smaller in *Foxp3^YFP-Cre/Y^Rosa26^Stat5b-CA/wt^* mice, while liver weight remained the same (**Figure 4C**). We then asked what led to the lean phenotype after STAT5B-CA expression in Treg cells. Continuous metabolic monitoring revealed a substantial reduction of food intake in *Foxp3^YFP-Cre/Y^Rosa26^Stat5b-CA/wt^* mice (**Figure 4D**), while energy expenditure (**Figure 4E**) and locomotor activity (**Figure 4F**) were both comparable to wildtype controls. Moreover, there was a tendency that fecal energy excretion was increased after Treg cell hyperactivation (**Figure 4G**). We then measured lipid content in the feces and found more lipids were excreted in *Foxp3^YFP-Cre/Y^Rosa26^Stat5b-CA/wt^* mice (**Figure 4H**). As expected, these HFD-fed but relatively lean *Foxp3^YFP-Cre/Y^Rosa26^Stat5b-CA/wt^* mice show much better glucose handling during the glucose tolerance test (**Figure 4I**). These data demonstrate that Treg cell expansion and activation stimulated by tonic STAT5 signaling restrain dietary fat intake/absorption and render mice resistant to diet-induced metabolic disturbances.

### Inducible STAT5 Activation in Treg Cells Improves Systemic Glucose Metabolism

**Figure 5:**
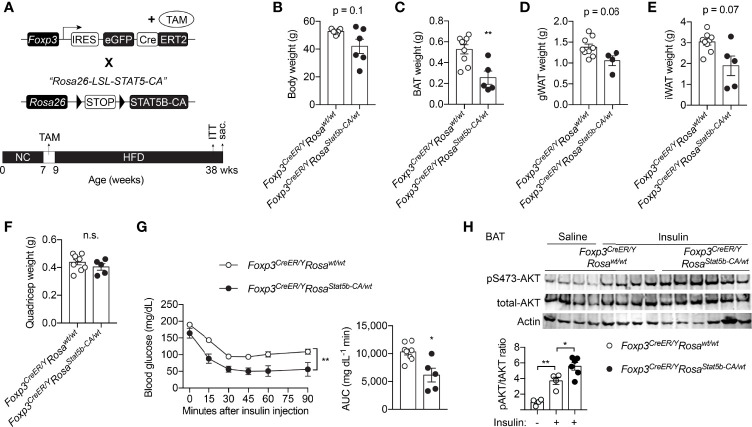
Inducible STAT5B-CA expression in Treg cells promotes metabolic adaptation to HFD. **(A)** Schematic view of the generation of tamoxifen-inducible, Treg cell-specific STAT5B-CA overexpression mice and dietary regimens to the mice. **(B)** Body weight of HFD-fed *Foxp3^eGFP-Cre-ERT2/Y^* (n = 6) and *Foxp3^eGFP-Cre-ERT2/Y^Rosa26^Stat5b-CA/wt^* (n = 6) mice. **(C–F)** Weight of BAT **(C)**, gonadal WAT **(D)**, inguinal WAT **(E)**, and quadricep muscle **(F)** of *Foxp3^eGFP-Cre-ERT2/Y^* (n = 9) and *Foxp3^eGFP-Cre-ERT2/Y^Rosa26^Stat5b-CA/wt^* (n = 5) mice. **(G)** Insulin tolerance test of HFD-fed *Foxp3^eGFP-Cre-ERT2/Y^* (n = 8) and *Foxp3^eGFP-Cre-ERT2/Y^Rosa26^Stat5b-CA/wt^* (n = 5). Area under curve (AUC) is shown to the right. **(H)** pS473-Akt and total-Akt immunoblotting of BAT protein lysates from *Foxp3^eGFP-Cre-ERT2/Y^* and *Foxp3^eGFP-Cre-ERT2/Y^Rosa26^Stat5b-CA/wt^* mice treated with saline or insulin for 30 min. Data are presented as mean ± SEM. n.s., not significant; *, p < 0.05; **, p < 0.01 by unpaired student’s t-test, one-way or two-way ANOVA.

Due to stochastic activity of the *Foxp3* promoter (43), constitutive overexpression of STAT5B-CA led to progressive splenomegaly and hematopoietic neoplasia in *Foxp3^YFP-Cre/Y^Rosa26^Stat5b-CA/wt^* mice (data not shown), similar to that after STAT5 activation in other hematopoietic compartments (44–46). We therefore used the inducible *Foxp3^eGFP-Cre-ERT2^* line to generate *Foxp3^eGFP-Cre-ERT2/Y^Rosa26^Stat5b-CA/wt^* male mice, fed them with tamoxifen-containing food to induce STAT5-CA expression in Treg cells, and challenged them with HFD feeding (**Figure 5A**). A trending reduction in weight gain could be observed in *Foxp3^eGFP-Cre-ERT2/Y^Rosa26^Stat5b-CA/wt^* mice (**Figure 5B**). When compared to their control counterparts, *Foxp3^eGFP-Cre-ERT2/Y^Rosa26^Stat5b-CA/wt^* mice possessed significantly less BAT (**Figure 5C**) and slightly smaller WAT (**Figure 5D, E**). No change in muscle mass was noticed (**Figure 5F**). Moreover, *Foxp3^eGFP-Cre-ERT2/Y^Rosa26^Stat5b-CA/wt^* animals also displayed much improved insulin tolerance (**Figure 5G**). Concomitantly, insulin signaling activity, reflected by AKT Ser473 phosphorylation, was augmented in BAT of *Foxp3^eGFP-Cre-ERT2/Y^Rosa26^Stat5b-CA/wt^* mice (**Figure 5H**). The more profound protection observed in the former constitutive model might stem from stronger and broader expression of STAT5B-CA. Nonetheless, using two complementary models, we demonstrate that STAT5 activation in Treg cells is sufficient to protect mice from diet-induced adiposity and insulin resistance.

### Treg Cell Activation Suppresses Lipid Uptake by Adipose Tissue

**Figure 6:**
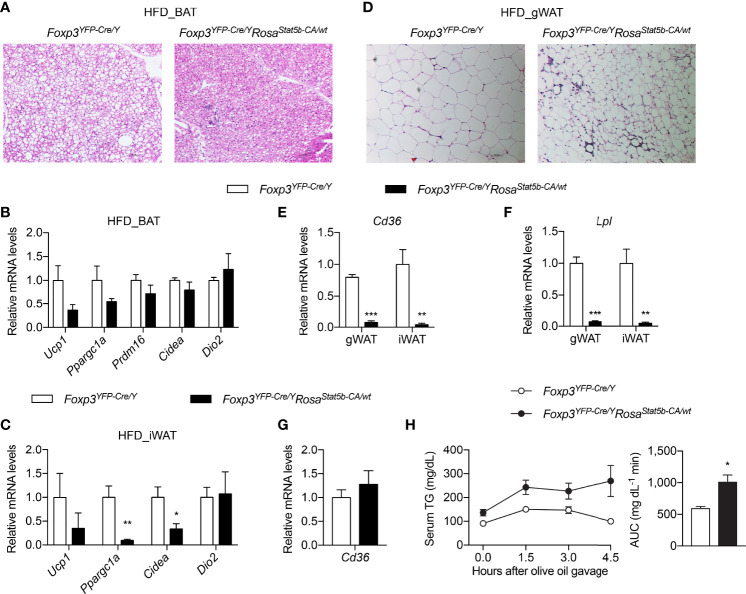
Thermogenesis and lipid metabolism in *Foxp3^YFP-Cre/Y^Rosa26^Stat5b-CA/wt^* mice. **(A)** BAT histology of HFD-fed *Foxp3^YFP-Cre/Y^* and *Foxp3^YFP-Cre/Y^Rosa26^Stat5b-CA/wt^* mice. **(B, C)** Expression of thermogenic genes in BAT **(B)** and iWAT **(C)** of HFD-fed *Foxp3^YFP-Cre/Y^* (n = 7) and *Foxp3^YFP-Cre/Y^Rosa26^Stat5b-CA/wt^* (n = 5) mice. **(D)** Gonadal WAT histology of HFD-fed *Foxp3^YFP-Cre/Y^* and *Foxp3^YFP-Cre/Y^Rosa26^Stat5b-CA/wt^* mice. **(E)** *Cd36* and **(F)** *Lpl* gene expression in WAT of HFD-fed *Foxp3^YFP-Cre/Y^* (n = 7) and *Foxp3^YFP-Cre/Y^Rosa26^Stat5b-CA/wt^* (n = 5) mice. **(G)** Expression of the *Cd36* gene in duodenum of HFD-fed *Foxp3^YFP-Cre/Y^* (n = 7) and *Foxp3^YFP-Cre/Y^Rosa26^Stat5b-CA/wt^* (n = 5) mice. **(H)** Oral lipid tolerance test of HFD-fed *Foxp3^YFP-Cre/Y^* (n = 9) and *Foxp3^YFP-Cre/Y^Rosa26^Stat5b-CA/wt^* (n = 6) mice. Serum triglyceride levels were determined. Area under curve (AUC) is shown to the right. Data are presented as mean ± SEM. *, p < 0.05; **, p < 0.01; ***, p < 0.001 by unpaired student’s t-test.

We then inquired into the causes of metabolic protection after STAT5-dependent Treg cell overactivation. We first interrogated the involvement of BAT thermogenesis. BAT histology clearly revealed smaller adipocytes and less lipid deposition in *Foxp3^YFP-Cre/Y^Rosa26^Stat5b-CA/wt^* mice (**Figure 6A**). However, the expression of thermogenic genes remained unchanged in BAT (**Figure 6B**), or even reduced in inguinal WAT (iWAT) (**Figure 6C**). This suggests that reduced weight gain by Treg cell activation was not a result of increased thermogenesis.

Not only was gWAT mass reduced in *Foxp3^YFP-Cre/Y^Rosa26^Stat5b-CA/wt^* mice (**Figures 4B, C**), but adipocyte size was also much smaller (**Figure 6D**). Crown-like structures were more evident in *Foxp3^YFP-Cre/Y^Rosa26^Stat5b-CA/wt^* gWAT (**Figure 6D**), suggesting active clearing of dying adipocytes by macrophages (47). Interestingly, the expression of both *Cd36* (**Figure 6E**) and *Lpl* (**Figure 6F**), two important genes required for cellular uptake of free fatty acid (48), were substantially ablated in gWAT and iWAT of *Foxp3^YFP-Cre/Y^Rosa26^Stat5b-CA/wt^* mice. There seemed to be no defect in dietary intake of lipid in the intestine, as intestinal *Cd36* expression was intact (**Figure 6G**) and blood triglyceride levels after an oral gavage of olive oil were even higher in *Foxp3^YFP-Cre/Y^Rosa26^Stat5b-CA/wt^* mice (**Figure 6H**). Taking in consideration of the elevated fecal lipid content (**Figure 4H**), we postulate that Treg cell activation suppresses lipid uptake into adipose tissues, leading to more energy excretion and less weight gain during HFD feeding.

### Treg Cell Activation Remodels Systemic Iron Metabolism

**Figure 7:**
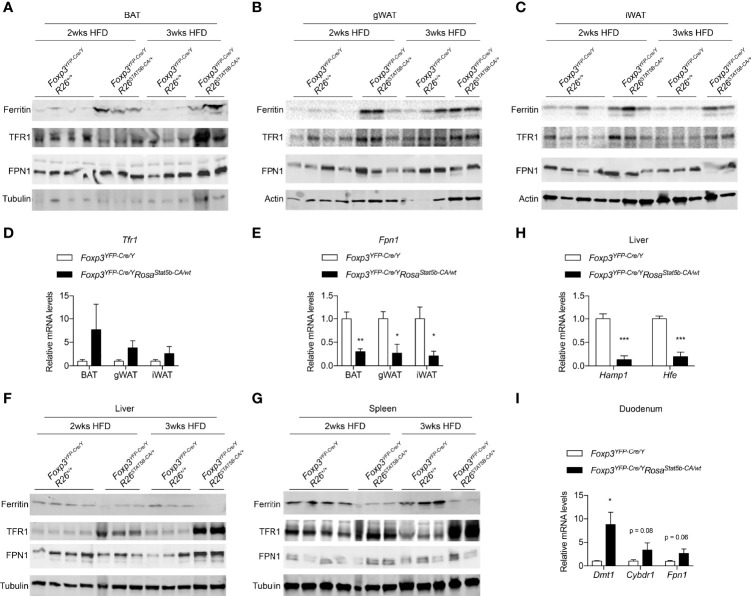
Iron metabolism modulated by Treg cell activation. **(A–C)** Immunoblotting of Ferritin, TFR1, FPN1 proteins in BAT **(A)**, gWAT **(B)**, and iWAT **(C)** of HFD-fed *Foxp3^YFP-Cre/Y^* and *Foxp3^YFP-Cre/Y^Rosa26^Stat5b-CA/wt^* mice. **(D, E)** Expression of the *Tfr1* **(D)** and *Fpn1* **(E)** genes in adipose tissues of HFD-fed *Foxp3^YFP-Cre/Y^* (n = 7) and *Foxp3^YFP-Cre/Y^Rosa26^Stat5b-CA/wt^* (n = 5) mice. **(F, G)** Immunoblotting of Ferritin, TFR1, FPN1 proteins in liver **(F)** and spleen **(G)** of HFD-fed *Foxp3^YFP-Cre/Y^* and *Foxp3^YFP-Cre/Y^Rosa26^Stat5b-CA/wt^* mice. **(H)** Expression of the *Hamp1* and *Hfe* genes in the liver of HFD-fed *Foxp3^YFP-Cre/Y^* (n = 7) and *Foxp3^YFP-Cre/Y^Rosa26^Stat5b-CA/wt^* (n = 5) mice. **(I)** Expression of the *Dmt*, *Cybdr1*, and *Fpn1* genes in the duodenum of HFD-fed *Foxp3^YFP-Cre/Y^* (n = 7) and *Foxp3^YFP-Cre/Y^Rosa26^Stat5b-CA/wt^* (n = 5) mice. Data are presented as mean ± SEM. *, p < 0.05; **, p < 0.01; ***, p < 0.001 by unpaired student’s t-test.

Iron is critically important for adipose tissue function and systemic metabolism. Dietary iron supplementation augments thermogenesis, reduces fat mass, and protects mice from diet-induced weight gain (49, 50). On the other hand, nonanemic iron deficiency impairs thermogenesis and promotes visceral obesity in mice (51). Lowering adipocyte iron uptake *via* genetic deletion of transferrin receptor 1 (TFR1) leads to impaired thermogenesis, increased insulin resistance, and inflammation (52). We then inquired if the metabolic protection after STAT5-dependent Treg cell activation involves iron. Ferritin is a protein complex that stores cellular iron and its protein levels were elevated in BAT, gWAT and iWAT of HFD-fed *Foxp3^YFP-Cre/Y^Rosa26^Stat5b-CA/wt^* mice, compared to Cre-only controls (**Figures 7A–C**), indicative of excess iron storage in fat tissues. In *Foxp3^YFP-Cre/Y^Rosa26^Stat5b-CA/wt^* adipose tissues, there was a trending increase in *Tfr1* gene expression (**Figure 7D**) and TFR1 protein levels (**Figures 7A–C**). Conversely, expression of the *Fpn1* gene (encoding iron export protein Ferroportin) was significantly downregulated (**Figure 7E**). In contrast to adipose tissue, both liver and spleen showed substantial reduction in Ferritin protein levels (**Figures 7F, G**), accompanied with increased TFR1 expression. The presumptive impairment in hepatic iron storage was correlated with reduced expression of iron-responsive *Hamp* and *Hfe* genes (**Figure 7H**), which encode Hepcidin and Homeostatic iron regulator, respectively. As a result of reduced Hepcidin, intestinal genes involved in iron uptake including *Dmt1*, *Cybrd1*, *Fn1* displayed varying degrees of upregulation (**Figure 7I**). Collectively, STAT5B-CA-mediated Treg cell overactivation redistributes the iron pool from liver and spleen to adipose tissues, which may contribute to the alleviation of metabolic stress associated with diet-induced obesity.

## Discussion

In effector Treg cells, protein O-GlcNAcylation induced by the TCR signaling, modifies and activates STAT5 for Treg cell homeostasis and function (38). In the study, we investigate whether and how the OGT-STAT5 regulatory axis dictates Treg cell function during metabolic adaptation to diet-induced obesity. Taking advantage of random X chromosome inactivation and mosaic OGT-deficiency in *Foxp3^YFP-Cre/wt^Ogt^F/F^* female mice, we establish that functional deficiency in about half the Treg cells renders animals more prone to diet-induced adiposity and insulin resistance. Our model is more advantageous than other Treg cell depletion models, such as the *Foxp3-DTR* mice (14), since the absence of systemic inflammation in *Foxp3^YFP-Cre/wt^Ogt^F/F^* females allowed us to perform long-term studies and avoided side effects and caveats of tissue inflammation (38). This is possibly also the reason why we observed obesity and thermogenic defects in *Foxp3^YFP-Cre/wt^Ogt^F/F^* mice, which have not been reported in other loss-of-function animals (14, 18, 22). We speculate that other heterozygous loss of Treg function models would show similar metabolic phenotypes as the *Foxp3^YFP-Cre/wt^Ogt^F/F^* mice, provided that systemic inflammation is avoided. In addition, we do not imply that Treg function in coping with obesity is specifically controlled by OGT, but rather OGT is essential for most if not all Treg functions, including their regulation of insulin sensitivity and systemic metabolism. For instance, we previously demonstrated that Blimp1^+^ effector Treg cells are ablated in OGT knockout mice (38). Similar to OGT knockout, Blimp1-deficient mice do not have effector Tregs (53). Recently, it was shown that Blimp1^+^ Tregs are also involved in the homeostasis of adipose tissue (54).

It is important to note that BAT harbors Treg cell populations that have shared and distinct gene signatures compared to WAT-resident Treg cells (55). Cold exposure or β3-adrenergic stimuli enhance Treg induction that is linked by a T cell-specific STAT6-Pten axis (56). When OGT is deficient in Treg cells, BAT insulin resistance and impaired thermogenesis could be observed, contributing to diet-induced obesity. Nonetheless, it is unclear if cold or β3-adrenergic activation could directly modulate the hexosamine biosynthetic pathway, OGT activity, and protein O-GlcNAcylation in Treg cells. Other work in our laboratory has revealed a pivotal role of O-GlcNAcylation on STAT6 signaling and type 2 immunity (57). It is warranted in the future to determine if OGT also regulates the STAT6-Pten axis in tissue-specific Treg cells and adipose tissue function.

IL-2 and IL-2R-dependent STAT5 activation are required for Treg cell development (5, 58). *Foxp3^YFP-Cre^*-mediated STAT5a/b knockout in male mice causes scurfy phenotypes and death by the age of 2 months (data not shown). We also generated mosaic *Foxp3^YFP-Cre/wt^Stat5a/b^F/F^* female mice avoiding fatal inflammation. However, we did not observe evident metabolic perturbations in these animals (data not shown), differently from *Foxp3^YFP-Cre/wt^Ogt^F/F^* females. It indicates that the metabolic effects of Treg cell O-GlcNAcylation might not be mediated just by STAT5, and other mechanisms such as STAT6 could compensate for the loss of STAT5. Nevertheless, STAT5B-CA overexpression is sufficient to booster Treg cell number and immunosuppression (10). Using these *Foxp3^YFP-Cre/Y^Rosa26^Stat5b-CA/wt^* mice, we demonstrate that Treg cell activation renders mice resistant to diet-induced weight gain and insulin resistance, in part by suppressing fatty acid uptake in adipose tissue and remodeling systemic iron metabolism. Intriguingly, no substantial changes in BAT thermogenesis were observed in STAT5B-CA overexpression mice, suggesting that STAT5-independent, but maybe STAT6-dependent mechanisms in Treg cells control adipose tissue lipolysis and thermogenesis (56).

While it has been widely accepted that Treg cells are important contributors to metabolic homeostasis, the regulatory mechanisms are not fully understood. Previous studies have revealed inflammation, lipolysis, and thermogenesis as tissue-specific targets of Treg cells. Our current study suggests two additional metabolic actions of Treg cells – fatty acid uptake and iron distribution. While intestinal lipid absorption is not perturbed, STAT5B-CA-driven Treg cell activation suppresses the expression of key genes involved in adipose fatty acid uptake – *Lpl* and *Cd36*. It would be important in the future to determine if the obesity protection in STAT5B-CA overexpression mice is dependent on LPL/CD36 downregulation. Meanwhile, the adipose iron pool seems to be expanded in STAT5B-CA overexpression, which we believe contributes to thermogenic activation and fat mass reduction. However, we are unclear whether parenchymal adipocytes or resident immune cells such as macrophages play a predominant role in the change in iron content. We also lack a mechanistic understanding of how Treg cells differentially regulate iron metabolism across different tissues (adipose vs. liver and kidney). Finally, it would be interesting to test if the suppression of lipid uptake is linked to iron redistribution in adipose tissues. In summary, our study has revealed new biology of Treg cells in metabolic regulation and the OGT-STAT5 axis as a target to harness Treg cell function for obesity and diabetes therapeutics.

## Data Availability Statement

The original contributions presented in the study are included in the article/**Supplementary Material**. Further inquiries can be directed to the corresponding author.

## Ethics Statement

The animal study was reviewed and approved by University of Minnesota.

## Author Contributions

ZZ and MF generated and analyzed STAT5B-CA mice. OS and KH did flow cytometry. BL analyzed OGT-KO mice. ZM helped with Western blot. H-BR conceived the project, analyzed data and wrote the manuscript. All authors contributed to the article and approved the submitted version.

## Funding

This work was supported by NIH grant R56 AI162791 to H-BR and R01 AI139420 to H-BR and MF.

## Conflict of Interest

The authors declare that the research was conducted in the absence of any commercial or financial relationships that could be construed as a potential conflict of interest.

## Publisher’s Note

All claims expressed in this article are solely those of the authors and do not necessarily represent those of their affiliated organizations, or those of the publisher, the editors and the reviewers. Any product that may be evaluated in this article, or claim that may be made by its manufacturer, is not guaranteed or endorsed by the publisher.

## References

[1] Josefowicz SZ Lu LF Rudensky AY. Regulatory T Cells: Mechanisms of Differentiation and Function. Annu Rev Immunol (2012) 30:531–64. doi: 10.1146/annurev.immunol.25.022106.141623 PMC6066374 22224781

[2] Sakaguchi S Yamaguchi T Nomura T Ono M. Regulatory T Cells and Immune Tolerance. Cell (2008) 133:775–87. doi: 10.1016/j.cell.2008.05.009 18510923

[3] Owen DL Mahmud SA Sjaastad LE Williams JB Spanier JA Simeonov DR. Thymic Regulatory T Cells Arise *via* Two Distinct Developmental Programs. Nat Immunol (2019) 20:195–205. doi: 10.1038/s41590-018-0289-6 30643267 PMC6650268

[4] Mahmud SA Manlove LS Schmitz HM Xing Y Wang Y Owen DL. Costimulation *via* the Tumor-Necrosis Factor Receptor Superfamily Couples TCR Signal Strength to the Thymic Differentiation of Regulatory T Cells. Nat Immunol (2014) 15:473–81. doi: 10.1038/ni.2849 PMC4000541 24633226

[5] Burchill MA Yang JY Vogtenhuber C Blazar BR Farrar MA. IL-2 Receptor Beta-Dependent STAT5 Activation is Required for the Development of Foxp3(+) Regulatory T Cells. J Immunol (2007) 178:280–90. doi: 10.4049/jimmunol.178.1.280 17182565

[6] Burchill MA Yang J Vang KB Moon JJ Chu HH Lio CW. Linked T Cell Receptor and Cytokine Signaling Govern the Development of the Regulatory T Cell Repertoire. Immunity (2008) 28:112–21. doi: 10.1016/j.immuni.2007.11.022 PMC2430111 18199418

[7] Lio CW Hsieh CS. A Two-Step Process for Thymic Regulatory T Cell Development. Immunity (2008) 28:100–11. doi: 10.1016/j.immuni.2007.11.021 PMC2248212 18199417

[8] Levine AG Arvey A Jin W Rudensky AY. Continuous Requirement for the TCR in Regulatory T Cell Function. Nat Immunol (2014) 15:1070–8. doi: 10.1038/ni.3004 PMC4205268 25263123

[9] Vahl JC Drees C Heger K Heink S Fischer JC Nedjic J. Continuous T Cell Receptor Signals Maintain a Functional Regulatory T Cell Pool. Immunity (2014) 41:722–36. doi: 10.1016/j.immuni.2014.10.012 25464853

[10] Chinen T Kannan AK Levine AG Fan X Klein U Zheng Y. An Essential Role for the IL-2 Receptor in Treg Cell Function. Nat Immunol (2016) 17:1322–33. doi: 10.1038/ni.3540 PMC5071159 27595233

[11] Fontenot JD Gavin MA Rudensky AY. Foxp3 Programs the Development and Function of CD4+CD25+ Regulatory T Cells. Nat Immunol (2003) 4:330–6. doi: 10.1038/ni904 12612578

[12] Hori S Nomura T Sakaguchi S. Control of Regulatory T Cell Development by the Transcription Factor Foxp3. Science (2003) 299:1057–61. doi: 10.1126/science.1079490 12522256

[13] Khattri R Cox T Yasayko SA Ramsdell F. An Essential Role for Scurfin in CD4+CD25+ T Regulatory Cells. Nat Immunol (2003) 4:337–42. doi: 10.1038/ni909 12612581

[14] Feuerer M Herrero L Cipolletta D Naaz A Wong J Nayer A. Lean, But Not Obese, Fat is Enriched for a Unique Population of Regulatory T Cells That Affect Metabolic Parameters. Nat Med (2009) 15:930–9. doi: 10.1038/nm.2002 PMC3115752 19633656

[15] Zeng Q Sun X Xiao L Xie Z Bettini M Deng T. A Unique Population: Adipose-Resident Regulatory T Cells. Front Immunol (2018) 9:2075. doi: 10.3389/fimmu.2018.02075 30323806 PMC6172295

[16] Kolodin D van Panhuys N Li C Magnuson AM Cipolletta D Miller CM. Antigen- and Cytokine-Driven Accumulation of Regulatory T Cells in Visceral Adipose Tissue of Lean Mice. Cell Metab (2015) 21:543–57. doi: 10.1016/j.cmet.2015.03.005 PMC4747251 25863247

[17] Vasanthakumar A Moro K Xin A Liao Y Gloury R Kawamoto S. The Transcriptional Regulators IRF4, BATF and IL-33 Orchestrate Development and Maintenance of Adipose Tissue-Resident Regulatory T Cells. Nat Immunol (2015) 16:276–85. doi: 10.1038/ni.3085 25599561

[18] Cipolletta D Feuerer M Li A Kamei N Lee J Shoelson SE. PPAR-Gamma is a Major Driver of the Accumulation and Phenotype of Adipose Tissue Treg Cells. Nature (2012) 486:549–53. doi: 10.1038/nature11132 PMC3387339 22722857

[19] Eller K Kirsch A Wolf AM Sopper S Tagwerker A Stanzl U. Potential Role of Regulatory T Cells in Reversing Obesity-Linked Insulin Resistance and Diabetic Nephropathy. Diabetes (2011) 60:2954–62. doi: 10.2337/db11-0358 PMC3198056 21911743

[20] Deng T Liu J Deng Y Minze L Xiao X Wright V. Adipocyte Adaptive Immunity Mediates Diet-Induced Adipose Inflammation and Insulin Resistance by Decreasing Adipose Treg Cells. Nat Commun (2017) 8:15725. doi: 10.1038/ncomms15725

[21] Li C DiSpirito JR Zemmour D Spallanzani RG Kuswanto W Benoist C. TCR Transgenic Mice Reveal Stepwise, Multi-Site Acquisition of the Distinctive Fat-Treg Phenotype. Cell (2018) 174:285–299 e12. doi: 10.1016/j.cell.2018.05.004 29887374 PMC6046274

[22] Matsumoto A Taniguchi K Takeda N Yamamura KI Arai S Miyazaki T. Inflammatory and Anti-Inflammatory States of Adipose Tissue in Transgenic Mice Bearing a Single TCR. Int Immunol (2017) 29:21–30. doi: 10.1093/intimm/dxx003 28182225 PMC5440033

[23] Bapat SP Myoung Suh J Fang S Liu S Zhang Y Cheng A. Depletion of Fat-Resident Treg Cells Prevents Age-Associated Insulin Resistance. Nature (2015) 528:137–41. doi: 10.1038/nature16151 PMC4670283 26580014

[24] Hart GW Housley MP Slawson C. Cycling of O-Linked Beta-N-Acetylglucosamine on Nucleocytoplasmic Proteins. Nature (2007) 446:1017–22. doi: 10.1038/nature05815 17460662

[25] Ruan HB Singh JP Li MD Wu J Yang X. Cracking the O-GlcNAc Code in Metabolism. Trends Endocrinol Metab (2013) 24:301–9. doi: 10.1016/j.tem.2013.02.002 PMC3783028 23647930

[26] Yang X Qian K. Protein O-GlcNAcylation: Emerging Mechanisms and Functions. Nat Rev Mol Cell Biol (2017) 18:452–65. doi: 10.1038/nrm.2017.22 PMC5667541 28488703

[27] Chang YH Weng CL Lin KI. O-GlcNAcylation and its Role in the Immune System. J BioMed Sci (2020) 27:57. doi: 10.1186/s12929-020-00648-9 32349769 PMC7189445

[28] Hanover JA Krause MW Love DC. Bittersweet Memories: Linking Metabolism to Epigenetics Through O-GlcNAcylation. Nat Rev Mol Cell Biol (2012) 13:312–21. doi: 10.1038/nrm3334 22522719

[29] Ruan HB Dietrich MO Liu ZW Zimmer MR Li MD Singh JP. O-GlcNAc Transferase Enables AgRP Neurons to Suppress Browning of White Fat. Cell (2014) 159:306–17. doi: 10.1016/j.cell.2014.09.010 PMC4509746 25303527

[30] Ruan HB Han X Li MD Singh JP Qian K Azarhoush S. O-GlcNAc Transferase/Host Cell Factor C1 Complex Regulates Gluconeogenesis by Modulating PGC-1alpha Stability. Cell Metab (2012) 16:226–37. doi: 10.1016/j.cmet.2012.07.006 PMC3480732 22883232

[31] Ruan HB Nie Y Yang X. Regulation of Protein Degradation by O-GlcNAcylation: Crosstalk With Ubiquitination. Mol Cell Proteomics (2013) 12:3489–97. doi: 10.1074/mcp.R113.029751 PMC3861702 23824911

[32] Hardiville S Hart GW. Nutrient Regulation of Signaling, Transcription, and Cell Physiology by O-GlcNAcylation. Cell Metab (2014) 20:208–13. doi: 10.1016/j.cmet.2014.07.014 PMC4159757 25100062

[33] Ruan HB Ma Y Torres S Zhang B Feriod C Heck RM. Calcium-Dependent O-GlcNAc Signaling Drives Liver Autophagy in Adaptation to Starvation. Genes Dev (2017) 31:1655–65. doi: 10.1101/gad.305441.117 PMC5647936 28903979

[34] Whelan SA Lane MD Hart GW. Regulation of the O-Linked Beta-N-Acetylglucosamine Transferase by Insulin Signaling. J Biol Chem (2008) 283:21411–7. doi: 10.1074/jbc.M800677200 PMC2490780 18519567

[35] Martinez MR Dias TB Natov PS Zachara NE. Stress-Induced O-GlcNAcylation: An Adaptive Process of Injured Cells. Biochem Soc Trans (2017) 45:237–49. doi: 10.1042/BST20160153 PMC6492270 28202678

[36] Zhao M Ren K Xiong X Cheng M Zhang Z Huang Z. Protein O-GlcNAc Modification Links Dietary and Gut Microbial Cues to the Differentiation of Enteroendocrine L Cells. Cell Rep (2020) 32:108013. doi: 10.1016/j.celrep.2020.108013 32783937 PMC7457433

[37] Zhao M Xiong X Ren K Xu B Cheng M Sahu C. Deficiency in Intestinal Epithelial O-GlcNAcylation Predisposes to Gut Inflammation. EMBO Mol Med (2018) 10(8):e8736. doi: 10.15252/emmm.201708736 29941542 PMC6079539

[38] Liu B Salgado OC Singh S Hippen KL Maynard JC Burlingame AL. The Lineage Stability and Suppressive Program of Regulatory T Cells Require Protein O-GlcNAcylation. Nat Commun (2019) 10:354. doi: 10.1038/s41467-019-08300-3 30664665 PMC6341091

[39] Wang Q Tang J Jiang S Huang Z Song A Hou S. Inhibition of PPARgamma, Adipogenesis and Insulin Sensitivity by MAGED1. J Endocrinol (2018) 239:167–80. doi: 10.1530/JOE-18-0349 30121577

[40] Huang Z Ruan HB Xian L Chen W Jiang S Song A. The Stem Cell Factor/Kit Signalling Pathway Regulates Mitochondrial Function and Energy Expenditure. Nat Commun (2014) 5:4282. doi: 10.1038/ncomms5282 24999927

[41] Kraus D Yang Q Kahn BB. Lipid Extraction From Mouse Feces. Bio Protoc (2015) 5(1):e1375. doi: 10.21769/bioprotoc.1375 PMC4838042 27110587

[42] Tschop MH Speakman JR Arch JR Auwerx J Bruning JC Chan L. A Guide to Analysis of Mouse Energy Metabolism. Nat Methods (2012) 9:57–63. doi: 10.1038/nmeth.1806 PMC3654855 22205519

[43] Bittner-Eddy PD Fischer LA Costalonga M. Cre-loxP Reporter Mouse Reveals Stochastic Activity of the Foxp3 Promoter. Front Immunol (2019) 10:2228. doi: 10.3389/fimmu.2019.02228 31616418 PMC6763954

[44] Burchill MA Goetz CA Prlic M O'Neil JJ Harmon IR Bensinger SJ. Distinct Effects of STAT5 Activation on CD4+ and CD8+ T Cell Homeostasis: Development of CD4+CD25+ Regulatory T Cells Versus CD8+ Memory T Cells. J Immunol (2003) 171:5853–64. doi: 10.4049/jimmunol.171.11.5853 14634095

[45] Pham HTT Maurer B Prchal-Murphy M Grausenburger R Grundschober E Javaheri T. STAT5BN642H is a Driver Mutation for T Cell Neoplasia. J Clin Invest (2018) 128:387–401. doi: 10.1172/JCI94509 29200404 PMC5749501

[46] Maurer B Nivarthi H Wingelhofer B Pham HTT Schlederer M Suske T. High Activation of STAT5A Drives Peripheral T-Cell Lymphoma and Leukemia. Haematologica (2020) 105:435–47. doi: 10.3324/haematol.2019.216986 PMC7012494 31123029

[47] Murano I Barbatelli G Parisani V Latini C Muzzonigro G Castellucci M. Dead Adipocytes, Detected as Crown-Like Structures, are Prevalent in Visceral Fat Depots of Genetically Obese Mice. J Lipid Res (2008) 49:1562–8. doi: 10.1194/jlr.M800019-JLR200 18390487

[48] Goldberg IJ Eckel RH Abumrad NA. Regulation of Fatty Acid Uptake Into Tissues: Lipoprotein Lipase- and CD36-Mediated Pathways. J Lipid Res (2009) 50 Suppl:S86–90. doi: 10.1194/jlr.R800085-JLR200 PMC2674753 19033209

[49] Romero AR Mu A Ayres JS. Fat Specific Adipose Triglyceride Lipase is Necessary for Iron-Mediated Lipolysis and Lipid Mobilization in Response to Negative Energy Balance. bioRxiv (2021) 2021:08. doi: 10.1101/2021.08.05.455308 PMC8899412 35265813

[50] Kitamura N Yokoyama Y Taoka H Nagano U Hosoda S Taworntawat T. Iron Supplementation Regulates the Progression of High Fat Diet Induced Obesity and Hepatic Steatosis *via* Mitochondrial Signaling Pathways. Sci Rep (2021) 11:10753. doi: 10.1038/s41598-021-89673-8 34031430 PMC8144192

[51] Yook JS Thomas SS Toney AM You M Kim YC Liu Z. Dietary Iron Deficiency Modulates Adipocyte Iron Homeostasis, Adaptive Thermogenesis, and Obesity in C57BL/6 Mice. J Nutr (2021) 151:2967–75. doi: 10.1093/jn/nxab222 PMC8485911 34383942

[52] Li J Pan X Pan G Song Z He Y Zhang S. Transferrin Receptor 1 Regulates Thermogenic Capacity and Cell Fate in Brown/Beige Adipocytes. Adv Sci (Weinh) (2020) 7:1903366. doi: 10.1002/advs.201903366 32596110 PMC7312276

[53] Cretney E Xin A Shi W Minnich M Masson F Miasari M. The Transcription Factors Blimp-1 and IRF4 Jointly Control the Differentiation and Function of Effector Regulatory T Cells. Nat Immunol (2011) 12:304–11. doi: 10.1038/ni.2006 21378976

[54] Beppu LY Mooli RGR Qu X Marrero GJ Finley CA Fooks AN. Tregs Facilitate Obesity and Insulin Resistance *via* a Blimp-1/IL-10 Axis. JCI Insight (2021) 6(3):e140644. doi: 10.1172/jci.insight.140644 PMC7934851 33351782

[55] Medrikova D Sijmonsma TP Sowodniok K Richards DM Delacher M Sticht C. Brown Adipose Tissue Harbors a Distinct Sub-Population of Regulatory T Cells. PloS One (2015) 10:e0118534. doi: 10.1371/journal.pone.0118534 25714366 PMC4340926

[56] Kalin S Becker M Ott VB Serr I Hosp F Mollah MMH. A Stat6/Pten Axis Links Regulatory T Cells with Adipose Tissue Function. Cell Metab (2017) 26:475–492 e7. doi: 10.1016/j.cmet.2017.08.008 28877454 PMC5627977

[57] Zhao M Ren K Xiong X Xin Y Zou Y Maynard JC. Epithelial STAT6 O-GlcNAcylation Drives a Concerted Anti-Helminth Alarmin Response Dependent on Tuft Cell Hyperplasia and Gasdermin C. Immunity (2022) 55:623–638 e5. doi: 10.1016/j.immuni.2022.03.009 35385697 PMC9109499

[58] Yao Z Kanno Y Kerenyi M Stephens G Durant L Watford WT. Nonredundant Roles for Stat5a/b in Directly Regulating Foxp3. Blood (2007) 109:4368–75. doi: 10.1182/blood-2006-11-055756 PMC1885496 17227828

