# Supplementary material for: An OGT-STAT5 Axis in Regulatory T Cells Controls Energy and Iron Metabolism
Source: Front Immunol. 2022 Jul 8;13:874863. doi: 10.3389/fimmu.2022.874863 (PMC9304952; doi:10.3389/fimmu.2022.874863)
Supplement: Supplementary file 1 [file Image_1.pdf]

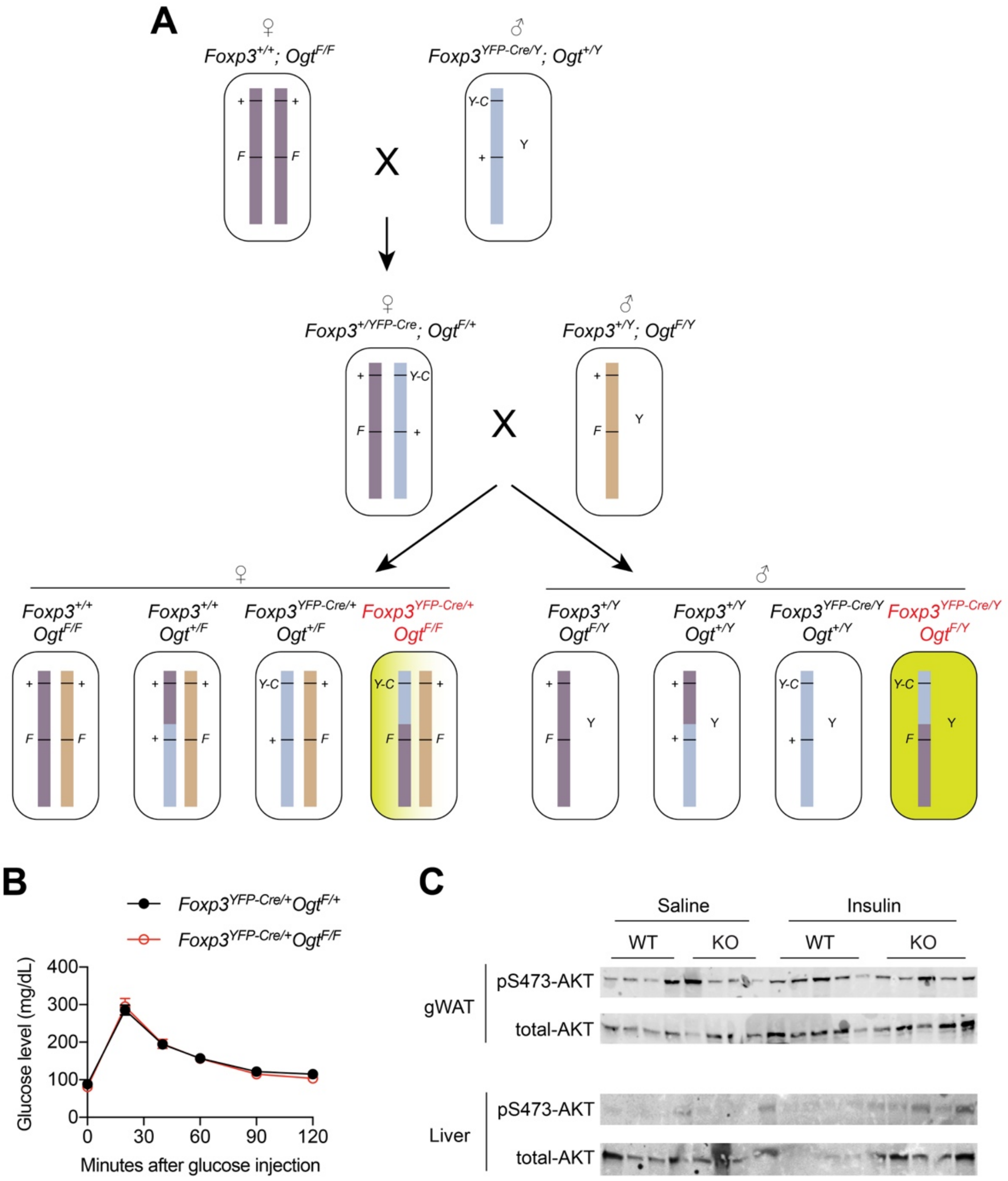

Figure S1. Related to Figure 1.

(A) Schematic diagram showing mating strategy to generate Treg cell-specific OGT knockout mice.

(B) Glucose tolerance test of 3-month-old, NC-fed *Foxp3*$^{YFP\text{-}Cre/wt}$*Ogt*$^{F/+}$ (n = 18) and *Foxp3*$^{YFP\text{-}Cre/wt}$*Ogt*$^{F/F}$ (n = 15) female mice.

(C) pS473-Akt and total-Akt immunoblotting of gonadal WAT and liver protein lysates from *Foxp3*$^{YFP\text{-}Cre/wt}$*Ogt*$^{F/+}$ and *Foxp3*$^{YFP\text{-}Cre/wt}$*Ogt*$^{F/F}$ female mice treated with saline or insulin for 30 min.
